# Cortical surface morphometry changes following combined translingual neural stimulation with the portable neuromodulation stimulator and physical therapy in patients with mild-to-moderate traumatic brain injury: a pilot study

**DOI:** 10.3389/fnins.2026.1975764

**Published:** 2026-10-07

**Authors:** Jiancheng Hou, Thomas Hosseini, Veena A. Nair, Yuri Danilov, Kurt Kaczmarek, Beth Meyerand, Mitchell Tyler, Vivek Prabhakaran

**Affiliations:** 1 Research Center for Cross-Straits Cultural Development, Fujian Normal University, Fuzhou, China; 2 Department of Radiology, School of Medicine and Public Health, University of Wisconsin-Madison, Madison, WI, United States

**Keywords:** cortical surface, dynamic gait index, sensory organization test, translingual neural stimulation, traumatic brain injuries

## Abstract

**Background:**

Traumatic brain injury (TBI) often causes persistent balance and gait deficits, especially in mild-to-moderate TBI (mmTBI). Physical therapy is standard but often limited as a standalone treatment. Translingual neural stimulation (TLNS) is an emerging neuromodulation approach combining cranial nerve stimulation with targeted exercise to promote neuroplasticity and recovery. Its effects on cortical surface morphometry in mmTBI remain uninvestigated.

**Methods:**

This secondary analysis used data from a completed clinical trial (NCT02158494). Nine chronic mmTBI patients underwent a two-week intervention combining TLNS (PoNS®) with targeted exercise training; no untreated, physical-therapy-only, or sham group was included. Balance and gait were assessed via the Sensory Organization Test (SOT) and Dynamic Gait Index (DGI) before and after the two-week in-lab phase. High-resolution T1-weighted MRI was acquired at both time points. Cortical thickness (CT), fractal dimensionality (FD), gyrification index (GI), and sulcal depth (SD) were analyzed using CAT12 with non-parametric permutation testing and TFCE correction.

**Results:**

SOT and DGI scores improved significantly; due to the single-group design, these pre–post changes cannot be attributed to TLNS, physical therapy, or their combination. Widespread pre-post CT increases occurred in bilateral frontal, temporal, parietal, and cingulate regions. FD showed increases (precuneus, insula, and superior frontal gyrus) and decreases (lateral occipital, inferior temporal gyri). GI decreased in the left lingual gyrus; SD showed bidirectional changes. Exploratory correlations linked greater behavioral gains with smaller CT increases in right inferior temporal gyrus and right superior parietal lobule, and smaller FD increases in left precuneus; these were underpowered (*N* = 9), uncorrected, and hypothesis-generating only. Morphometric changes in either direction cannot be assumed to reflect beneficial neuroplasticity.

**Conclusion:**

In this exploratory, single-group pilot study, cortical surface morphometric changes accompanied balance and gait improvements after combined TLNS plus physical therapy in mm TBI. Without a control group, causality cannot be determined. Findings are preliminary and support randomized, sham-controlled trials, ideally with a physical-therapy-only arm.

## Introduction

Traumatic brain injury (TBI) occurs when an external force rapidly accelerates the skull, and can lead to cognitive, physical, emotional, and social impairments (McDonald, 2013). In mild-to-moderate traumatic brain injury (mmTBI), balance disorders are present in 30–65% of patients (Peterson and Greenwald, 2015). While physical therapy is the current standard of care, it has a limited effect when used as the sole treatment method (Han et al., 2011).

Translingual neural stimulation (TLNS) is a novel interventional approach that combines non-invasive trigeminal and facial nerve stimulation with physical therapy, with the aim of restoring balance and gait (Danilov et al., 2015). TLNS can be employed via the Portable Neuromodulation Stimulator (PoNS®, Helius Medical Technologies, Newtown, Pennsylvania, United States), a medical apparatus that transmits electrical stimulation to the tongue. The excitation of the facial and trigeminal nerves elicits a flow of nerve impulses to the cerebellum and the brainstem, inducing metabolic changes in related brain structures (Wildenberg et al., 2011a, 2011b, 2013). Studies indicate that TLNS promotes neurostimulation-induced neuroplasticity (e.g., the pons, cerebellum, and brainstem in brain) in mmTBI patients without causing tissue damage, leading to symptom reduction overall (Danilov et al., 2015; Wildenberg et al., 2010, 2011b, 2013).

A brain morphometry study showed that when TLNS treatment was administered in concert with physical therapy to mmTBI patients, gray matter volume (GMV) increases were observed in the temporal and cerebellum regions that are involved in automatic processing of gait, balance, motor control and visual-motion. Concurrently, decreased GMV was observed in the frontal and occipital regions, suggesting a reduction in automatic processing and more conscious/effortful processing of gait, balance, motor control and vision. These results highlight the role of TLNS in promoting brain plasticity and the positive therapeutic effects of TLNS treatment (Hou et al., 2022; Tyler et al., 2019).

However, volumetric analysis has certain limitations, such as insufficient sensitivity to brain surface folding (Jin et al., 2018; Lemaitre et al., 2012). In addition, the intensity contrast of magnetic resonance signals between gray and white matter leads to an underestimation of their actual rate (Jin et al., 2018; Kochunov et al., 2005; Lemaitre et al., 2012). Cortical surface morphometry provides a more detailed assessment, incorporating parameters such as the cortical thickness (CT), fractal dimension (FD), gyrification index (GI) and sulcal depth (SD) (Hirakawa et al., 2016; Trost et al., 2013). CT measures the distance between the points on the pial and white matter boundaries of the neocortex, along with the GM morphological difference (Hirakawa et al., 2016; Seiger et al., 2018). However, CT is limited to the cortex so it cannot examine non-cortical regions (Bermudez et al., 2009). FD reflects how the brain structure fits to space constraints (Yotter et al., 2011b) and is used to investigate brain complexity (Di Ieva et al., 2015; Di Ieva et al., 2014; Madan and Kensinger, 2017), providing insights that GMV and CT alone cannot capture (Chen et al., 2020; Madan and Kensinger, 2017; Zhang et al., 2008). GI examines the level of local cortical folding that relates to the integrality between subcortical and cortex circuits (Li et al., 2021). SD is based on the Euclidean distance between the pial and outer surface (Li et al., 2021; Yun et al., 2013) and is generated from the changes in gray and white matter in the cerebral cortex as well as of subcortical structures (Im et al., 2008; Jin et al., 2018; Kim et al., 2008; Kochunov et al., 2008). Given the fact that SD is not limited to the gray and white matter and is more sensitive to the complicated folding of cerebral surface, it has been a relatively new way to investigate brain structure (Jin et al., 2018).

Emerging evidence from structural neuroimaging suggests that these cortical surface morphometric indices are functionally relevant to balance and gait control. In healthy adults, a recent study using the split-belt treadmill paradigm showed that greater cortical gyrification in the pre/postcentral gyri was associated with less step length asymmetry, while greater sulcal depth in the insula was associated with more step length asymmetry during gait adaptation (Fettrow et al., 2023). These findings align with the functional roles of the pre/postcentral gyri in sensorimotor integration and the insula in body awareness and interoception during locomotion. CT has also been linked to gait domains in older adults: poorer gait pace was independently associated with thinner cortex in motor, sensory, visuospatial attention, and cognitive regions, including the primary motor cortex, postcentral gyrus, lateral occipital cortex, and fusiform gyrus (Weiniger and Schilaty, 2024). Additionally, regional CT has been associated with gait variability, with smaller thickness in frontal and temporal regions correlating with greater variability in step time and step width (Jayakody et al., 2020).

In TBI populations specifically, a recent study of individuals with chronic TBI reported that FD of gray matter within the default mode, sensorimotor, and frontoparietal networks was positively correlated with balance performance, suggesting that cortical structural complexity may serve as a biomarker of sensorimotor function after injury (Alivar et al., 2025). FD has also shown utility in TBI for capturing white matter complexity and predicting executive function outcomes beyond conventional diffusion metrics (Esteban et al., 2007). Together, these findings provide a rationale for examining whether TLNS-induced changes in CT, FD, GI, and SD parallel behavioral improvements in balance and gait.

The present study is a secondary analysis of imaging and behavioral data from a previously completed TLNS trial in mmTBI (NCT02158494) (Hou et al., 2022). While our prior work in this cohort reported gray matter volume and resting-state functional connectivity changes, the current analysis focuses on cortical surface morphometry (CT, FD, GI, SD) and its association with balance/gait outcomes—measures not previously examined in this cohort. Despite the potential for TLNS to induce neuroplastic changes, and the emerging links between cortical surface morphometry and gait/balance function, there are no studies to date that have investigated surface-based morphometric changes following TLNS intervention in mmTBI patients, nor directly linked such changes to balance/gait outcomes in this population. Therefore, the current study applied the measurements of CT, FD, GI and SD, all of which mutually disambiguate and complement each other’s weaknesses, to assess cortical surface changes from pre to post-intervention following the combined TLNS + physical therapy intervention and their associations with behavioral improvements. Because all participants received both components and no untreated, physical-therapy-only, or sham-stimulation arm was included, the present design cannot isolate the contribution of the stimulation component.

## Methods

### Study design and timeline of the present secondary analysis

The present study is a secondary analysis of imaging and behavioral data from a previously completed investigator-initiated clinical trial (NCT02158494), which was a single-arm, open-label trial of translingual neural stimulation (TLNS) using the Portable Neuromodulation Stimulator (PoNS®; Helius Medical Technologies, Newtown, Pennsylvania, United States) combined with physical therapy in chronic mild-to-moderate traumatic brain injury (mmTBI). The parent trial consisted of two sequential phases: (1) a 2-week in-lab phase, during which participants received twice-daily in-lab TLNS plus a targeted physical exercise training program, with at-home training during the intervening weekend; and (2) a subsequent 12-week at-home phase, during which participants returned to the clinic weekly for a single session of retraining and exercise progression and participated in periodic retesting. In the parent trial, behavioral assessments [Sensory Organization Test (SOT) and Dynamic Gait Index (DGI)] and T1-weighted structural MRI were collected at baseline, at the end of the 2-week in-lab phase, and at 3-week intervals during the 12-week at-home/follow-up phase.

The present secondary analysis is restricted to the baseline and end-of-2-week-in-lab assessments, because these were the two time points at which both behavioral (SOT, DGI) and usable T1-weighted structural MRI data were available for all nine participants. Data from the 12-week at-home phase and from the 3-week-interval follow-up assessments were not included in the present cortical surface morphometry, behavioral, or correlation analyses. Accordingly, all pre–post changes reported in this manuscript refer specifically to changes from baseline to the end of the 2-week in-lab TLNS plus physical therapy phase, and do not reflect the full 14-week parent-trial protocol or longer-term follow-up. A study timeline and flow diagram illustrating the parent-trial phases, assessment time points, and the data included in the present analysis is provided in Figure 1.

**Figure 1:**
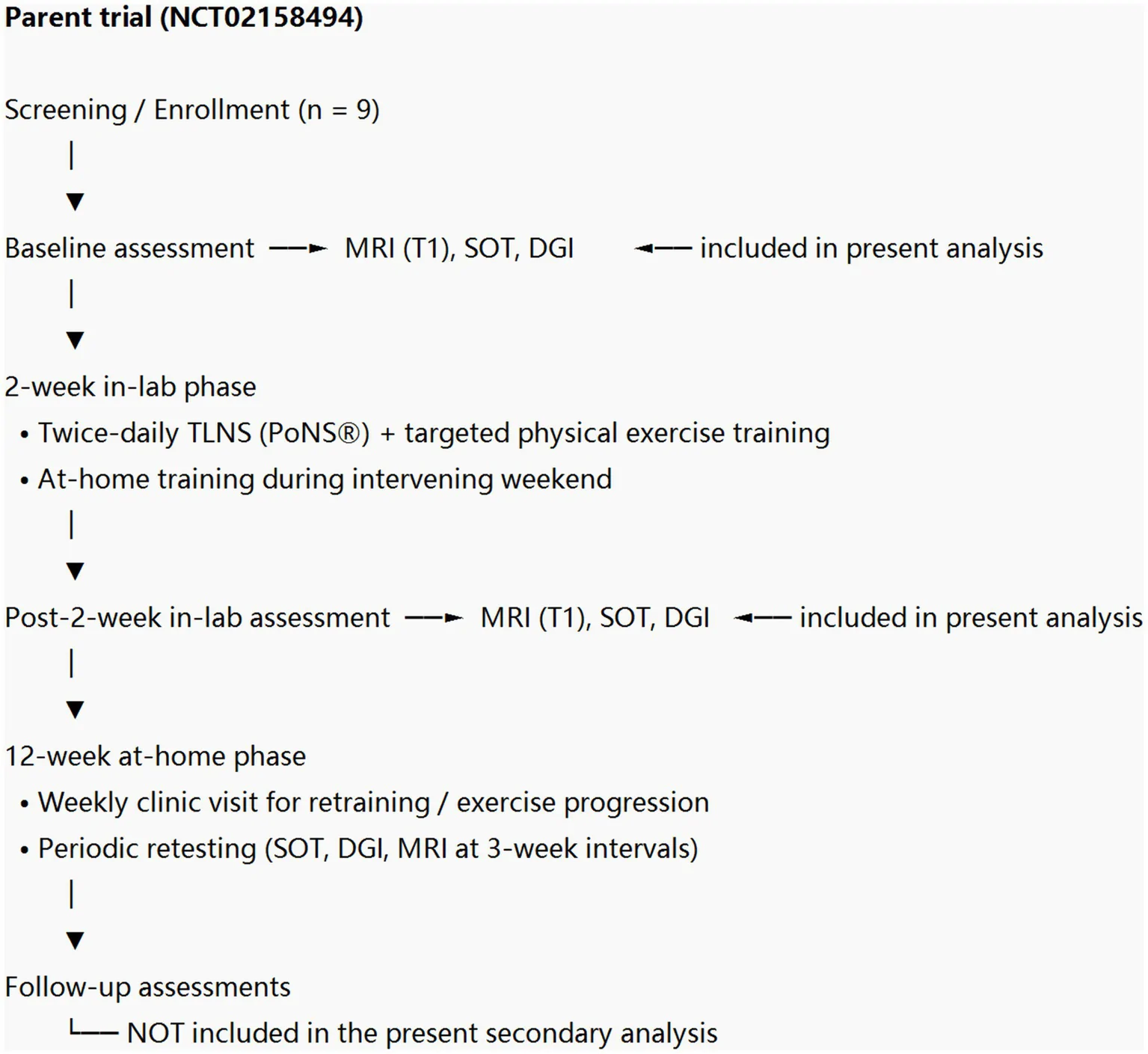
Study timeline of the parent trial (NCT02158494) and scope of the present secondary analysis. The parent trial comprised a 2-week in-lab phase of twice-daily TLNS using PoNS® combined with a targeted physical exercise training program, followed by a 12-week at-home phase with weekly retraining sessions and periodic retesting. The present secondary analysis includes only the baseline and end-of-2-week-in-lab assessments, because these were the two time points with both behavioral (SOT, DGI) and usable T1-weighted MRI data for all nine participants. Data from the 12-week at-home phase and follow-up retesting intervals (dashed box) were not analyzed in the present study.

All participants in the parent trial received active TLNS plus physical therapy; the parent trial did not include an untreated, physical-therapy-only, or sham-stimulation arm. Therefore, the present design cannot isolate the contribution of the stimulation component from that of physical therapy, their combination, or other factors associated with repeated assessment. This limitation is acknowledged in the Discussion.

### Participants

TBI severity was classified according to the VA/DoD Clinical Practice Guidelines (Management of Concussion/mTBI Working Group, 2009), which define mild TBI (GCS 13–15, LOC ≤ 30 min, AOC ≤ 24 h, PTA ≤ 1 day) and moderate TBI (GCS 9–12, LOC > 30 min and <24 h, PTA > 1 day and <7 days) as distinct categories. The term “mild-to-moderate TBI (mmTBI)” is used in the present study as a descriptive term for the severity spectrum represented in this cohort, consistent with the original trial’s eligibility criteria (Hou et al., 2022). Individual-level GCS, LOC, AOC, and PTA data were not retained in the trial dataset; therefore, retrospective classification of each participant as mild versus moderate was not possible. This limitation is acknowledged in the Discussion.

The nine participants are the same mmTBI cohort reported in Hou et al. (2022). They represent the participants from the parent trial who completed the 2-week in-lab phase and for whom usable pre and post-intervention T1-weighted structural MRI scans were available. Participant flow for the present secondary analysis was as follows: of the nine participants enrolled in NCT02158494, all completed the 2-week in-lab TLNS plus physical therapy phase, and all had usable pre and post-intervention T1-weighted structural MRI scans. No participants were excluded from the present analysis because of excessive head motion, incomplete brain coverage, failed preprocessing, or a missing post-intervention scan, yielding the nine participants analyzed here. These nine participants represent all parent-trial participants eligible for the present MRI-based cortical surface morphometry analysis. The behavioral and MRI data analyzed here were acquired as part of the original trial protocol (NCT02158494) and are the same data from the same participant cohort reported in Hou et al. (2022). No additional imaging data were acquired for the present secondary analysis, and no additional participants were recruited.

These nine participants with mmTBI (43 to 62 years old; mean age = 53.11 ± 6.60; 3 males and 6 females) were included in the study. All participants had sustained a mmTBI at least 1 year before enrollment, had previously participated in physical therapy, had reached functional stability in their recovery, and scored 16 points below normal on an age-adjusted Sensory Organization Test (SOT) composite score. The mmTBI diagnoses were made according to the guidelines established by the Veterans Affairs/Department of Defense (Hou et al., 2022; Management of Concussion/mTBI Working Group, 2009). All participants received the combined intervention (TLNS using PoNS® plus a targeted physical exercise training program); no untreated, physical-therapy-only, or sham-stimulation comparison group was included, so the effects of the stimulation component, physical therapy, and their combination cannot be separated. The study was approved by the Institutional Review Board (IRB) at University of Wisconsin–Madison School of Medicine and Public and all participants were recruited on a voluntary basis and signed the approved consent form before participation in the study.

All participants were able to walk independently for at least 20 minutes, had access to a treadmill outside of clinic, and had no changes in their medications for at least 3 months prior to participation. Exclusion criteria included oral health issues, non-removable metal orthodontic devices, chronic infectious disease, oral cavity piercings that could interfere with TLNS use, uncontrolled hypertension, diabetes, neurological disorders unrelated to their primary diagnosis, penetrating head injury, craniotomy, refractory subdural hematoma, and a history of treatment for cancer other than basal cell carcinoma within the past year. Additional exclusion criteria included a history of seizures, the use of lower extremity biomechanical prosthetics, and a “severe” score in any of the attention, memory or executive functions categories on the Cognitive Linguistic Quick Test (CLQT) (Tyler et al., 2019).

### Intervention

The combined intervention focused on the recovery of balance and gait and consisted of twice-daily in-lab training sessions over 2 weeks. All participants received TLNS using PoNS® together with a targeted physical exercise training program designed to improve motor coordination and mobility. All participants received both components; no untreated, physical-therapy-only, or sham-stimulation comparison group was included, so the effects of the stimulation component, physical therapy, and their combination cannot be separated. Participants returned to the clinic weekly during a subsequent 12-week at-home phase for a single session of retraining and exercise progression, and participated in periodic retesting.

Behavioral and imaging assessment metrics analyzed in the present secondary analysis—the SOT, DGI, and T1-weighted structural MRI—were collected at baseline, at the end of the two-week in-lab phase, and at three-week intervals during follow-up. The SOT and DGI are described in the Behavioral testing section, and MRI acquisition parameters are described in the MRI data acquisition section. The full original trial assessment battery is reported in Hou et al. (2022).

TLNS was administered using the PoNS device (version 2.2). The PoNS device delivers electrical stimulation through a mouthpiece containing an electrode array positioned on the anterior-dorsal surface of the tongue. The array comprises 143 gold-plated electrodes distributed across 16 independent stimulation channels, with 9 electrodes activated simultaneously at any given moment (each active electrode surrounded by return-path electrodes). Each electrode is approximately 1.5 mm in diameter, creating an activation area of approximately 1.77 mm^2^ per electrode and a total activated area of approximately 15.9 mm^2^ (0.16 cm^2^) at any moment. The stimulation waveform is pulsed biphasic (unbalanced) and rectangular, with a primary depolarizing phase duration of 60 μs and a pulse duration of 312.5 μs. Pulses are delivered in triplets at 200 Hz intervals, with channel pulses stimulating different tongue locations at 3.2 kHz, repeating every 50 Hz. Maximum output voltage is 17.5 V at 500 Ω and 20 V at 5 kΩ; maximum output current is 3.5 mA at 500 Ω (per channel) and 440 μA at 5 kΩ. Stimulation intensity was individually adjusted to a comfortable level according to each participant’s tolerance, consistent with the standard PoNS Therapy protocol (PoNS device technical specifications, FDA *De Novo* DEN200050).

### Behavioral testing

All participants completed the Sensory Organization Test (SOT) and the Dynamic Gait Index (DGI) at baseline and after 2 weeks of twice-daily in-lab TLNS interventions as part of the behavioral battery. SOT is an objective and automated assessment of sensory-motor integration that evaluates the relative contributions of visual, somatosensory, and vestibular components to balance. The SOT outcome analyzed here is the composite Equilibrium Score derived from the six standard SOT conditions (C1–C6); this composite score is the conventional primary SOT outcome and was the metric used to determine eligibility in the parent trial. Condition-level SOT scores were not analyzed in the present study, because the parent trial used the composite score as its primary SOT outcome and because condition-level analyses would be substantially underpowered at *N* = 9. DGI is a clinician-scored evaluation that assesses eight facets of gait, with scores ranging from 0 (worst) to 24 (normal). A change of three points is typically considered clinically significant (Hou et al., 2022). The behavioral data analyzed here—SOT and DGI scores at baseline and at the end of the 2-week in-lab phase—are the same data from the same participant cohort that were previously reported in Hou et al. (2022). No additional behavioral data were collected for the present secondary analysis.

### MRI data acquisition

T1-weighted structural MRI scans were obtained using a 3 T MRI GE750 scanner (GE Healthcare, Waukesha, Wisconsin, United States) before and immediately after the two-week in-lab TLNS intervention. Imaging parameters for the MPRAGE sequence were: TR/TE/θ = 8,132 ms/3.18 ms/12°, FOV = 100 × 100 mm, in-plane resolution = 1 × 1 mm^2^, and slice thickness = 1 mm. During scanning, participants lay supine with their heads secured using foam pads to minimize movement. They were instructed to keep their heads still, close their eyes, and remain relaxed but awake. All participants confirmed that they remained awake throughout the scan.

### Data preprocessing

The Computational Anatomy Toolbox (CAT, version 12; http://www.neuro.uni-jena.de/cat/), which is a plug-in software based on Statistical Parametric Mapping (SPM, version 12, https://www.fil.ion.ucl.ac.uk/spm/software/spm12/) and integrated into MATLAB (MathWorks, version R2020a), was used for the T1-weighted MRI data preprocessing. The CAT12 is not only a precise analysis for GMV (Farokhian et al., 2017; Yuksel et al., 2018) but can also fully automate for analyzing surface morphometry (Zhuang et al., 2017). The data preprocessing with CAT12 consisted of bias-field correction, skull-stripping, and alignment to the Montreal Neurological Institute (MNI) template to classify GM, white matter (WM) and cerebrospinal fluid (CSF) as well as spatial normalization with the Diffeomorphic Anatomical Registration Through Exponentiated Lie Algebra (DARTEL) registration (Kurth et al., 2015; Yuksel et al., 2018; Zhuang et al., 2017). A spherical harmonic method (Yotter et al., 2011a) was then employed to reparametrize the cortical surface mesh based on an algorithm that reduces the area aberration (Yotter et al., 2011c) to repair topological flaws (Chen et al., 2020; Yotter et al., 2011a, 2011c). Four complementary surface-based morphometric measures were calculated by CAT12: (1) CT, defined as the distance between the pial and white matter surfaces; (2) FD, reflecting the complexity of the cortical surface mesh; (3) GI, indexing the degree of local cortical folding; and (4) SD, derived from the Euclidean distance between the pial surface and the outer convex hull. These measures were selected because they capture distinct and complementary aspects of cortical surface morphology—thickness, complexity, folding, and sulcal geometry—and together compensate for the limited sensitivity of volumetric measures to cortical surface folding (Jin et al., 2018; Lemaitre et al., 2012). Detailed definitions and the rationale for each measure are provided in the Cortical Surface Morphometry subsection. Given the absence of prior surface-based morphometric studies of TLNS in mmTBI, this analysis was exploratory, and no *a priori* directional hypotheses were specified for individual measures.

### Cortical surface morphometry

Cortical surface reconstruction and morphometric estimation were performed using the CAT12 surface pipeline, implemented in SPM12 and MATLAB (R2020a). Following tissue segmentation and DARTEL normalization, each participant’s cortical surface was reconstructed from the T1-weighted image using a projection-based thickness algorithm (Dahnke et al., 2013), and the central surface was generated in native space before being resampled to the fsaverage template for inter-subject alignment. Four surface-based measures were then extracted: (1) CT was computed as the distance between the pial surface and the white matter surface, estimated via a tissue-level projection scheme that accounts for partial volume information and sulcal blurring (Dahnke et al., 2013; Seiger et al., 2018). CT values were sampled at each vertex of the central surface. (2) FD was estimated using a spherical harmonic reconstruction of the cortical surface, in which the surface mesh is reparameterized and its complexity quantified as the fractal dimension of the reconstructed mesh (Yotter et al., 2011b). FD reflects the degree to which the cortical surface fills the available space within a given radius and is sensitive to local surface complexity beyond what CT or gray matter volume capture (Di Ieva et al., 2014; Madan and Kensinger, 2017). (3) GI was calculated as the ratio of the integrated cortical surface area to the area of the corresponding outer hull surface within a spherical region of interest (Luders et al., 2006) and implemented in CAT12 (Li et al., 2021). GI therefore indexes the degree of local cortical folding. (4) SD was derived from the Euclidean distance between each vertex on the pial surface and the corresponding point on the outer surface (i.e., the convex hull), such that positive values indicate sulcal regions and values near zero indicate gyral crowns (Im et al., 2008; Jin et al., 2018; Yun et al., 2013). SD is generated from the geometric relationship between gray matter, white matter, and subcortical structures and is more sensitive to complex cortical folding than volumetric measures alone. All four measures were smoothed on the surface with a Gaussian kernel: 15 mm FWHM for CT and 20 mm FWHM for FD, GI, and SD, following the CAT12 recommendations for thickness versus folding-based surface parameters. The smoothed measures were then entered into the statistical analysis described below.

### Statistical analysis

Demographic and clinical characteristics were summarized descriptively; pre to post-intervention differences in behavioral outcomes (SOT and DGI, see Table 1) were assessed using paired *t-tests*. Cortical surface morphometry measures (CT, FD, GI, and SD) were analyzed using non-parametric permutation tests within CAT12. Threshold-Free Cluster Enhancement (TFCE) was applied to correct for familywise error (FWE) at *p* < 0.05, with 5,000 permutations. This TFCE correction controls the FWE across all cortical vertices within each surface measure; the TFCE-corrected *p* values reported in Table 2 therefore already account for multiple comparisons across the cortical surface within each measure. Only clusters exceeding 100 vertices were reported. Surface regions were labeled using the Desikan–Killiany (DK40) atlas. Results were visualized using CAT12. The CAT12/SPM permutation output provides two complementary maps: (i) a signed statistic map, in which positive values indicate post > pre and negative values indicate post < pre, and (ii) a separate TFCE-corrected *p*-value map with values bounded between 0 and 1. In the present figures, we display the signed statistic map masked by the TFCE-corrected significance threshold, so that the color bar represents the direction and magnitude of the pre–post change rather than a *p* value. TFCE-corrected *p* values for each significant cluster are reported in Table 2. The figures should therefore be interpreted as directional pre–post maps, not as *p*-value maps.

**Table 1:** Behavioral outcomes at pre and post-interventions.

Measure	Pre-intervention (mean ± SD)	Post-intervention (mean ± SD)	*t*_(8)_	*p*
SOT	43.000 ± 15.215	64.556 ± 14.723	2.742	0.028
DGI	19.444 ± 4.333	20.889 ± 3.756	2.855	0.024

SOT = Sensory Organization Test; DGI = Dynamic Gait Index. Values are group means ± standard deviations (*N* = 9). *p*-values are from two-tailed paired-samples *t*-tests (baseline vs. post-intervention). Because the parent trial was single-arm and open-label, these pre–post changes cannot be attributed to the stimulation component, to physical therapy, or to their combination. SOT composite scores are age-adjusted Equilibrium Scores (range 0–100); DGI scores range from 0 (worst) to 24 (normal), with a 3-point change considered clinically meaningful. These behavioral data are the same as those previously reported in Hou et al. (2022).

**Table 2:** Cortical surface changes between post vs. pre-interventions.

Group differences	Regions	Cluster size	TFCE-corrected *p*	*p* corrected across 4 outcomes (Bonferroni)	Significant or not after cross-outcome correction
Thickness (left hemisphere)					
Post > Pre	Superior frontal gyrus	122	0.002	0.008	Yes
	Superior frontal gyurs	145	0.004	0.016	Yes
	Caudal middle frontal gyrus	398	0.002	0.008	Yes
	Medial orbitofrontal gyrus	133	0.033	0.132	No
	Precentral gyrus	371	0.002	0.008	Yes
	Postcentral gyrus	287	0.002	0.008	Yes
	Superior temporal gyrus	479	0.016	0.064	No
	Inferior temporal gyrus	148	0.020	0.080	No
	Superior parietal lobule	373	0.002	0.008	Yes
	Inferior parietal lobule	101	0.002	0.008	Yes
	Inferior parietal lobule	110	0.033	0.132	No
	Pars opercularis gyrus	100	0.002	0.008	Yes
	Supramarginal gyrus	209	0.016	0.064	No
	Supramarginal gyrus	382	0.002	0.008	Yes
	Fusiform	443	0.020	0.080	No
Thickness (right hemisphere)					
Post > Pre	Superior frontal gyrus	1,413	0.002	0.008	Yes
	Medial orbitofrontal gyrus	283	0.002	0.008	Yes
	Lateral orbitofrontal gyrus	283	0.002	0.008	Yes
	Rostral middle frontal gyrus	1,130	0.002	0.008	Yes
	Superior temporal gyrus	424	0.002	0.008	Yes
	Middle temporal gyrus	424	0.002	0.008	Yes
	Inferior temporal gyrus	424	0.002	0.008	Yes
	Superior parietal lobule	1,130	0.002	0.008	Yes
	Inferior parietal lobule	1,695	0.002	0.008	Yes
	Lateral occipital gyrus	424	0.002	0.008	Yes
	Caudal anterior cingulate gyrus	141	0.002	0.008	Yes
	Posterior cingulate gyrus	283	0.002	0.008	Yes
	Precentral gyrus	424	0.002	0.008	Yes
	Postcentral gyrus	706	0.002	0.008	Yes
	Pars triangularis gyrus	424	0.002	0.008	Yes
	Pars opercularis gyrus	424	0.002	0.008	Yes
	Paracentral gyrus	283	0.002	0.008	Yes
	Supramarginal gyrus	1,413	0.002	0.008	Yes
	Precuneus	989	0.002	0.008	Yes
	Fusiform	283	0.002	0.008	Yes
	Bankssts gyrus	283	0.002	0.008	Yes
	Isthmuscingulate gyrus	283	0.002	0.008	Yes
	Insula	182	0.008	0.032	Yes
Fractal dimensionality (left hemisphere)					
Post > Pre	Precuneus	224	0.018	0.072	No
	Superior frontal gyrus	149	0.014	0.056	No
Post < Pre	Pericalcarine	191	0.004	0.016	Yes
	Lateral occipital gyrus	153	0.008	0.032	Yes
	Inferior temporal gyrus	132	0.010	0.040	Yes
Fractal dimensionality (right hemisphere)					
Post > Pre	Superior frontal gyrus	128	0.029	0.116	No
	Rostral middle frontal gyrus	299	0.002	0.008	Yes
	Caudal middle frontal gyrus	183	0.002	0.008	Yes
	Insula	105	0.014	0.056	No
Gyrification (left hemisphere)					
Post < Pre	Lingual gyrus	224	0.021	0.084	No
Sulcal depth (left hemisphere)					
Post > Pre	Middle temporal gyrus	101	0.004	0.016	Yes
	Superior temporal gyrus	137	0.004	0.016	Yes
	Lateral occipital gyrus	116	0.004	0.016	Yes
	Supramarginal gyrus	215	0.014	0.056	No
Post < Pre	Entorhinal gyrus	178	0.002	0.008	Yes
	Parahippocampal gyrus	152	0.002	0.008	Yes
Sulcal depth (right hemisphere)					
Post > Pre	Superior frontal gyrus	828	0.002	0.008	Yes
	Rostral middle frontal gyrus	534	0.002	0.008	Yes
	Lateral orbitofrontal gyrus	138	0.002	0.008	Yes
	Parsorbitalis gyrus	103	0.002	0.008	Yes
	Superior temporal gyrus	131	0.031	0.124	No
	Supramarginal gyrus	131	0.004	0.016	Yes
	Insula	149	0.004	0.016	Yes

The multiple comparison correction was used with non-parametric permutations (*n* = 5,000) and threshold-free cluster enhancement (TFCE) correction threshold *p* < 0.05 after 5,000 permutations. All entries represent directional pre–post changes from baseline to the end of the 2-week in-lab phase. The *p*-values reported in this table are TFCE-corrected p values for each cluster; they are distinct from the signed statistic displayed in the color bars of Figure 2, which represents the direction and magnitude of the pre–post change. These changes should not be interpreted as evidence of beneficial or detrimental neuroplasticity.

Because four primary surface outcomes were examined (CT, FD, GI, and SD), an additional Bonferroni correction across the four outcomes was applied (*α* = 0.05/4 = 0.0125). For each cluster, we report both the TFCE-corrected *p* value (within-measure FWE correction) and the Bonferroni-adjusted *p* value across the four outcomes [*p*_Bonf = min (TFCE-corrected p × 4, 1)], together with an indicator of whether the cluster remained significant after this cross-outcome correction (see Table 2). Clusters that survived TFCE correction within a measure but did not survive the cross-outcome Bonferroni correction are described as exploratory in the Results and Discussion. We note that Bonferroni is the most conservative approach; an FDR-based correction (Benjamini–Hochberg) across the four outcomes would yield a less stringent threshold and would leave more clusters significant. We chose the more conservative Bonferroni correction to reduce the risk of false-positive findings given the small sample size.

Pearson correlation analyses were conducted between cortical surface morphometric changes (post minus pre) and behavioral performance changes (post minus pre) in SOT and DGI. All morphometric and behavioral change scores used in these analyses were computed from the baseline and end-of-2-week-in-lab assessments; data from the 12-week at-home phase and follow-up intervals were not included. To reduce the number of comparisons and focus on regions showing intervention-related morphometric changes, correlation analyses were restricted to cortical regions that exhibited significant group-level morphometric changes in the TFCE permutation analysis (see Table 3). For each significant region, the change in that morphometric measure was correlated with the change in SOT and with the change in DGI, separately. This resulted in a total of 122 correlation tests (CT: 38 regions × 2 behavioral measures; FD: 9 regions × 2; GI: 1 region × 2; SD: 13 regions × 2). In the brain regions where significant differences were observed in CT, FD, GI and SD, we extracted the specific brain region values, and then the correlation analysis between each morphometry measure (post minus pre-training) and behavioral test (post minus pre-training) was used to perform correlation analysis based on Pearson’s correlation and set to correct *p* < 0.05 with SPSS version 23. Given the exploratory nature of these analyses and the small sample size, the significant correlations reported in Table 2 should be interpreted as hypothesis-generating. Because Pearson correlations are sensitive to outliers and distributional assumptions and are underpowered at this sample size, Spearman’s rank-order correlations were computed alongside Pearson *r* for all brain–behavior relationships reported in Table 3. Both coefficients are reported so that readers can assess the robustness of the findings to the choice of correlation method. No correction for multiple comparisons across the 122 exploratory tests was applied; the *p* values are therefore uncorrected and should be interpreted descriptively. Correlations were considered significant at *p* < 0.05.

**Table 3:** Correlations between cortical surface measures and behavioral testing.

Cortical surface measures	Regions	*r_(9)_/p*	DGI (Pearson/Spearman)	SOT (Pearson/Spearman)
Cortical thickness	Right inferior temporal gyrus	*r*_(9)_	−0.670/−0.683	
		*p*	0.048/0.042	
	Right superior parietal lobule	*r*_(9)_	−0.719/−0.733	
		*p*	0.029/0.025	
Fractal dimensionality	Left precuneus	*r*_(9)_		−0.737/−0.717
		*p*		0.023/0.030

SOT: Sensory organization test; DGI: Dynamic Gait Index. All correlated morphometric measures showed post > pre increases. Negative r values indicate that greater behavioral improvement was associated with a smaller post–pre increase. These analyses are exploratory, uncorrected for multiple comparisons across the 122 tests, and underpowered at *N* = 9; they should be interpreted as hypothesis-generating.

## Results

All findings below describe changes from pre to post-intervention following the combined TLNS plus physical therapy intervention and are restricted to the baseline and end-of-2-week-in-lab assessments; data from the 12-week at-home phase and follow-up assessments were not included in these analyses. Because of the single-group design, these changes cannot be attributed to the stimulation component, to physical therapy, to their combined effects, or to other factors associated with repeated assessment. In addition, changes in cortical surface morphometry are reported here as directional pre–post observations and should not be interpreted as evidence of beneficial or detrimental neuroplasticity (see Discussion).

### Behavior analysis

Because these behavioral results have been reported previously (Hou et al., 2022), they are presented here only to provide the context for the cortical surface morphometry findings and should be regarded as previously published; the novel contribution of the present manuscript is the surface-based morphometric analysis and its association with these behavioral outcomes. Paired *t*-tests revealed significant improvements in from pre-intervention to post-intervention in both the SOT and DGI (see Table 1). Individual and group-level behavioral changes are shown in Figure 2. For the SOT composite, scores increased from a baseline (pre-intervention) mean of 43.000 ± 15.215 (range 10–60) to a post-intervention mean of 64.556 ± 14.723 (range 40–84) (Figure 2A, individual trajectories; Figure 2B, group means with SD error bars). For the DGI, scores increased from a baseline mean of 19.444 ± 4.333 (range 10–24) to a post-intervention mean of 20.889 ± 3.756 (range 12–24) (Figure 2C, individual trajectories; Figure 2D, group means with SD error bars). Given the single-arm design, these improvements should be interpreted as pre–post changes rather than treatment-specific effects. As noted above, these behavioral data are restricted to the baseline and end-of-2-week-in-lab assessments; SOT and DGI data from the 12-week at-home phase and follow-up intervals were not analyzed here.

**Figure 2:**
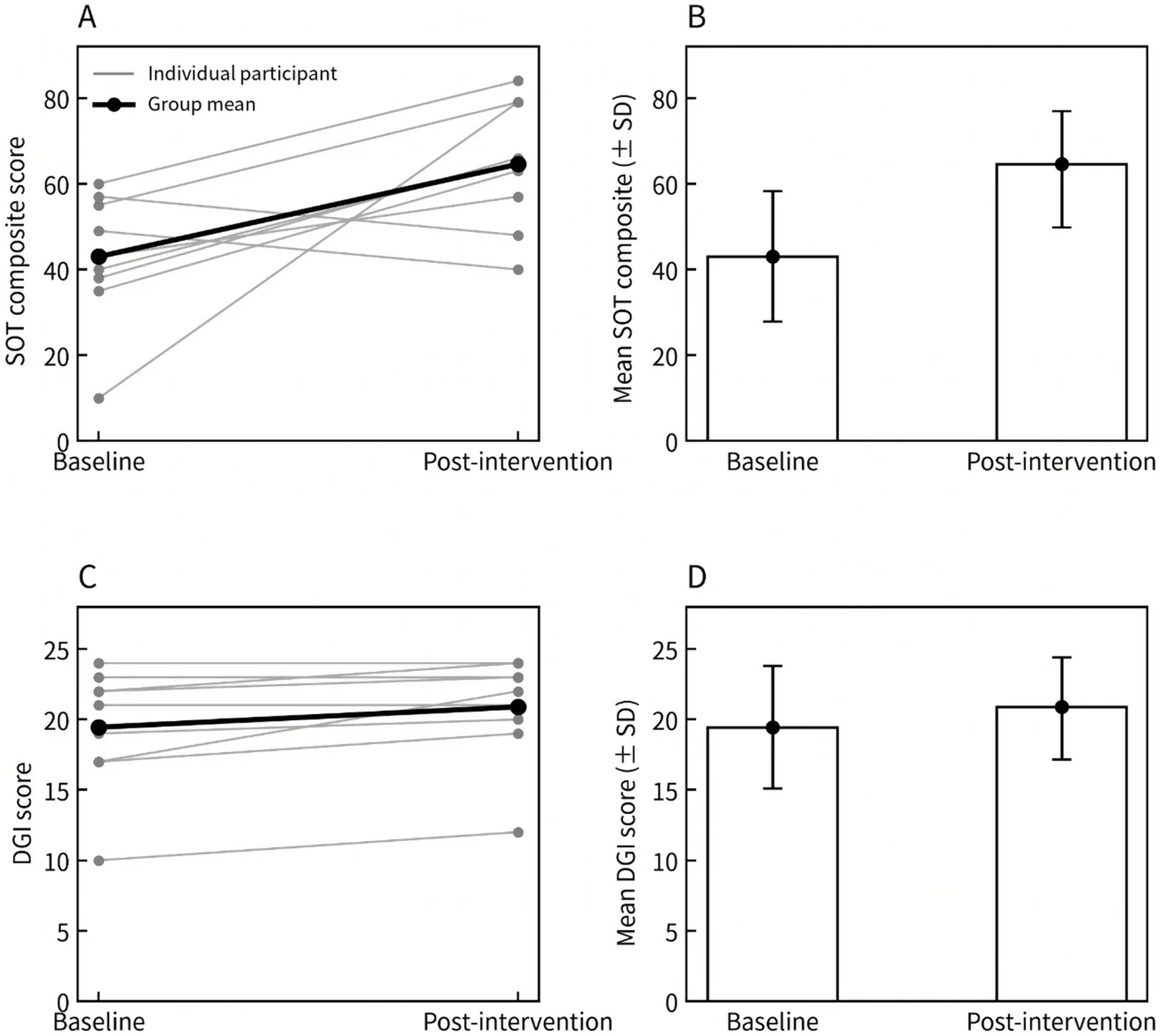
Individual and group-level behavioral changes from pre-intervention (baseline) to the end of the 2-week in-lab TLNS plus physical therapy phase. **(A)** Individual SOT score at baseline and post-intervention; lines connect the same participant. **(B)** Group mean SOT composite score with error bars (mean ± SD) and the paired *t*-test result. **(C)** Individual DGI score at baseline and post-intervention. **(D)** Group mean DGI score with error bars and the paired *t*-test result. These behavioral data are the same as those previously reported in Hou et al. (2022) and are shown here to provide the behavioral context for the cortical surface morphometry findings. Because the parent trial was single-arm, open-label, and included no untreated, physical-therapy-only, or sham-stimulation arm, these pre–post changes cannot be attributed to TLNS, to physical therapy, or to their combination.

### Cortical analysis

The cortical surface morphometry results below describe directional pre–post changes from baseline to the end of the 2-week in-lab phase; data from the 12-week at-home phase and follow-up assessments were not included in these analyses. Both increases and decreases in CT, FD, GI, and SD are reported. In the accompanying figures (see Figure 3), the color bar represents the signed test statistic (post—pre) from the non-parametric permutation analysis, masked by the TFCE-corrected significance threshold; positive values (red/yellow) indicate post > pre and negative values (blue) indicate post < pre. The color bar does not represent a *p* value; TFCE-corrected *p* values for each significant cluster are reported in Table 2. The direction of change does not by itself indicate functional benefit or harm, and these observations should not be equated with beneficial neuroplasticity; multiple biological and methodological sources can produce changes in either direction (see Discussion).

(1) CT

**Figure 3:**
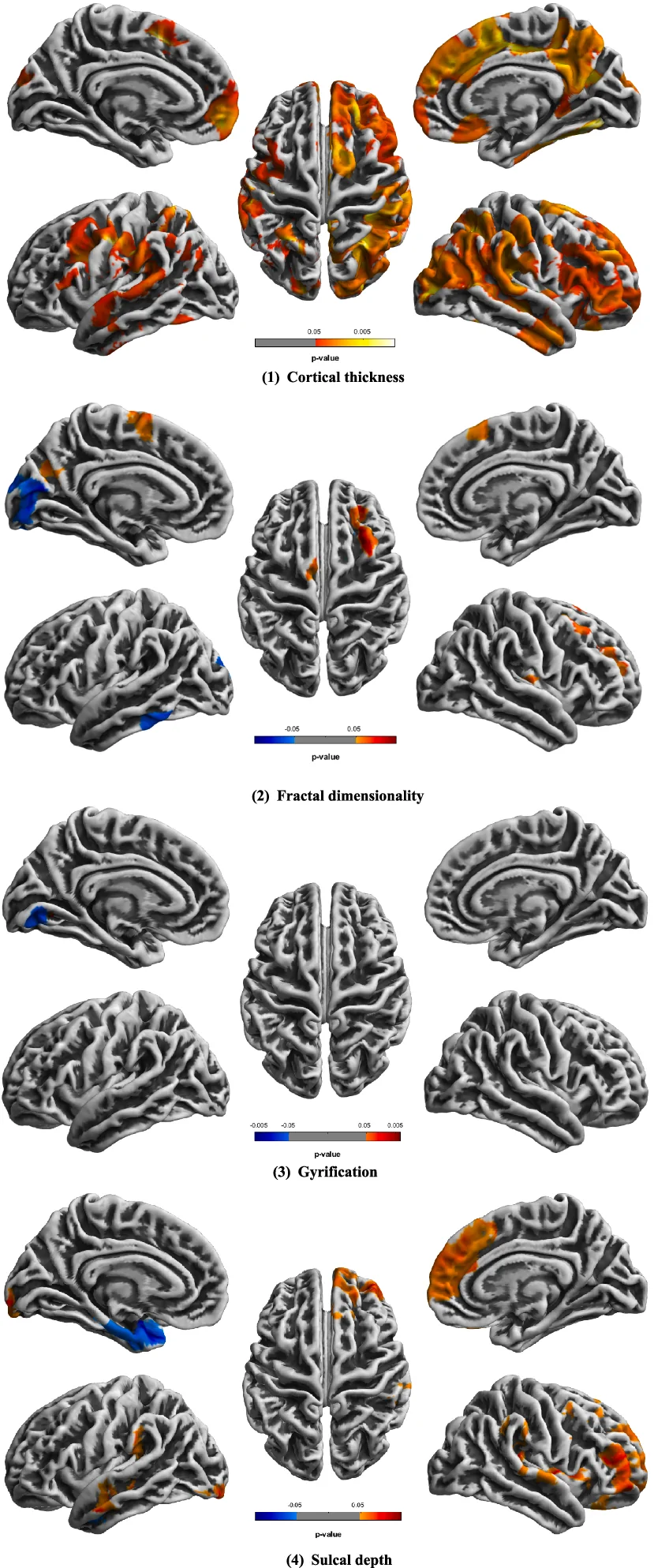
Cortical surface differences between post and pre-interventions following the combined TLNS plus physical therapy intervention. Maps display the signed test statistic (post—pre) from the non-parametric permutation analysis, masked by the TFCE-corrected significance threshold (*p* < 0.05, 5,000 permutations). In addition to the TFCE *p* < 0.05 threshold, a cluster extent threshold of >100 vertices was applied, and only clusters exceeding 100 vertices are displayed. Red/yellow indicates post > pre; blue indicates post < pre. The color bar represents the signed statistic (post—pre), not a *p* value; TFCE-corrected *p* values for each significant cluster are reported in Table 2. The left side of each panel indicates the left hemisphere. These maps display directional pre–post changes and should not be interpreted as evidence of beneficial or detrimental neuroplasticity. Because all participants received the combined intervention, these maps should not be interpreted as stimulation-specific effects.

Analysis showed significant pre-post increases in CT across multiple brain regions from pre-intervention to post-intervention. In the left hemisphere, increased CT was observed in the caudal middle frontal gyrus. In the right hemisphere, significant increases were found in the lateral orbitofrontal, rostral middle frontal, middle temporal, lateral occipital, caudal anterior cingulate, posterior cingulate, pars triangularis, paracentral, precuneus, bankssts, isthmus cingulate and insula gyri. Bilateral increases in CT were noted in the superior frontal, medial orbitofrontal, precentral, postcentral, superior temporal, inferior temporal, pars opercularis, supramarginal, fusiform gyri, superior parietal, and inferior parietal lobules (see Figure 3(1) and Table 2).

(2) FD

FD results indicated both increases and decreases following the combined TLNS plus physical therapy intervention. Significant increases in FD were seen in the left precuneus, right insula, rostral and caudal middle frontal gyri, and bilateral superior frontal gyri. In contrast, significant decreases were observed in the left pericalcarine, lateral occipital, and inferior temporal gyri (see Figure 3(2) and Table 2).

(3) GI

A single significant change was observed in GI, with a post-intervention decrease seen in left lingual gyrus (see Figure 3(3) and Table 2).

(4) SD

SD results also indicated both increases and decreases following the combined TLNS plus physical therapy intervention. Significant increases were seen in middle temporal and lateral occipital gyri in left hemisphere, the superior frontal, rostral middle frontal, lateral orbitofrontal, pars orbitalis and insula gyri in right hemisphere, and the superior temporal and supramarginal gyri bilaterally. In contrast, significant decreases were observed in the entorhinal and parahippocampal gyri in left hemisphere (see Figure 3(4) and Table 2).

### Correlation analysis

Exploratory correlation analysis between cortical morphometric changes and behavioral performance revealed significant relationships. CT increased from pre to post-intervention in the right inferior temporal gyrus and right superior parietal lobule, and FD increased in the left precuneus. The changes in CT in these two regions were negatively correlated with DGI improvement, and the change in FD in the left precuneus was negatively correlated with SOT improvement (see Table 3). That is, greater behavioral improvement was associated with a smaller post–pre increase in these measures. Because *N* = 9 yields low statistical power for Pearson correlations, Spearman’s rank-order correlations were computed alongside Pearson *r*; both coefficients were consistent in direction and significance for all three relationships. These correlations are exploratory and uncorrected for multiple comparisons across the 122 tests; the confidence intervals around these coefficients are wide, and the findings require replication in larger samples. Importantly, the negative direction of these correlations should not be interpreted as evidence of a single mechanism (e.g., “neural efficiency”), nor as evidence that smaller morphometric change is beneficial. Alternative and non-mutually exclusive explanations include baseline variance and regression-to-the-mean, individual differences in injury severity and chronicity, focal edema resolution versus structural remodeling, compensatory plasticity, partial volume and segmentation effects, test–retest measurement variability, and the small sample size (see Discussion).

## Discussion

In this exploratory, single-arm pilot analysis, statistically significant improvements in both SOT and DGI scores were observed from pre to post-intervention, accompanied by notable changes in cortical surface morphometry. All pre–post changes reported here refer specifically to changes from baseline to the end of the 2-week in-lab TLNS plus physical therapy phase; data from the 12-week at-home phase and follow-up assessments were not included in the present analysis, and the present study therefore cannot characterize the trajectory of morphometric or behavioral changes beyond the immediate post-2-week-in-lab period. The most important limitation of this study is the absence of an untreated, physical-therapy-only, or sham-stimulation comparison group. Consequently, it is impossible to determine whether the observed changes were attributable to the stimulation component, to physical therapy, to their combined effects, or to other factors associated with repeated assessment (e.g., practice effects, test–retest variability, and scanner/preprocessing variability). These pre–post changes therefore cannot be attributed specifically to TLNS, and alternative explanations—including naturally occurring longitudinal change, test–retest variability in cortical surface morphometry, practice effects on the SOT and DGI, and scanner and preprocessing-related variability—cannot be excluded. All findings reported here should be regarded as preliminary and hypothesis-generating. In addition, and importantly for the interpretation of the present results, changes in cortical surface morphometry in either direction cannot be assumed to represent beneficial neuroplasticity; the morphometric changes reported here should therefore be regarded as structural observations that paralleled behavioral improvement, not as evidence of a restorative or therapeutic mechanism.

Several key brain regions showed pre–post changes in cortical surface morphometry following the combined TLNS plus physical therapy intervention. Given the absence of a sham control, these changes should be interpreted as preliminary observations that may or may not be specifically related to TLNS. The postcentral gyrus, also known as the primary somatosensory cortex (S1) processes sensory information and facilitates goal-oriented behavior by delivering sensory information to the premotor cortex (Zastron et al., 2019). The supramarginal gyrus, part of the temporoparietal junction, integrates multimodal sensory inputs, including somatosensory and visual inputs (Bzdok et al., 2013; zu Eulenburg et al., 2012), and plays a role in visual-vestibular interactions (Pfeiffer et al., 2014; Zastron et al., 2019). The inferior parietal lobule, a component of the default mode and visuospatial network (Dixon et al., 2018; Zhang and Li, 2014), interacts with the nearby supramarginal gyrus and has been identified as a multisensory vestibular region (Dieterich and Brandt, 2018). Additionally, the precuneus, another region within the default mode network, is responsible for visual perception and imagery (Demorest et al., 2010; Schmithorst, 2005).

Other regions that exhibited cortical surface changes post-intervention include the superior frontal gyrus, which is responsible for motor control and visuospatial perception (Wang et al., 2017); the inferior temporal gyrus, which contributes to visual perception and imagery (Miyashita, 1993; Rolls et al., 2003); the superior parietal lobule, which is involved in visuo-spatial processing (Gaser and Schlaug, 2003); the postcentral gyrus, which plays a role in sensorimotor perception and multisensory integration (Loui et al., 2011); and the insula, which is associated with sensorimotor control and movement representation (Schulze et al., 2011). The observed changes in cortical surface morphometry may be consistent with altered sensorimotor, visual, and cognitive processing, but without a control condition they cannot be attributed to TLNS, nor can they be assumed to represent beneficial neuroplasticity.

In contrast, decreases in FD, GI and SD were observed in regions associated with visual processing (pericalcarine (Coullon et al., 2015), lateral occipital gyrus (Emberson et al., 2017), inferior temporal gyrus (Miyashita, 1993; Rolls et al., 2003), lingual gyrus (Gaab et al., 2006)), learning and memory (entorhinal gyrus (Amaral et al., 2014)), and visuospatial processing and memory [parahippocampal gyrus (Aminoff et al., 2013; Bohbot et al., 2015)]. The reason for these reductions remains unclear. They might reflect improved cognitive efficiency and automaticity, but this interpretation is speculative, and no causal attribution to TLNS can be made without a control condition. Alternatively—and non-mutually exclusively—decreases in FD, GI, and SD may reflect resolution of subtle focal edema, partial volume effects, tissue normalization, or measurement variability rather than atrophy, loss of complexity, or “efficiency.” Without a control group and without fluid-sensitive sequences (e.g., FLAIR) or diffusion imaging (e.g., DTI), these possibilities cannot be distinguished.

Exploratory correlation analysis revealed negative relationships between cortical surface morphometry and behavioral performance. In the regions showing significant correlations, CT and FD increased from pre to post-intervention (post > pre). The negative correlations indicate that greater behavioral improvement was associated with a smaller increase (i.e., a less pronounced post–pre increase) in CT in the right inferior temporal gyrus and right superior parietal lobule, and in FD in the left precuneus. The inferior temporal gyrus and precuneus, both involved in visual perception and imagery, as well as the superior parietal lobule, which plays a role in visuo-spatial processing, showed negative correlations with SOT and DGI scores. Although these findings could be interpreted as reflecting more efficient visual and spatial processing, several alternative and non-mutually exclusive mechanisms should be considered, and the present design cannot distinguish among them: (1) Baseline variance and regression-to-the-mean/ceiling effects. Participants with higher baseline cortical values or greater baseline symptom burden may have less room for further structural increase while still showing behavioral gains, which can produce negative brain–behavior correlations independent of any true “efficiency” mechanism. Similarly, individuals with lower baseline performance have more room to improve, which can induce negative correlations with change scores. (2) Individual differences in injury severity, chronicity, and reserve. Although all participants met mmTBI criteria, the cohort likely differed in injury severity, number and location of prior injuries, time since injury, and pre-existing cognitive/sensorimotor reserve. These differences could produce both smaller structural changes and larger behavioral gains in some individuals, again yielding negative correlations. (3) Focal edema resolution versus structural remodeling. Post–pre decreases in FD, GI, and SD in visual and limbic regions could reflect resolution of subtle focal edema, partial volume effects, or normalization of initially altered tissue rather than atrophy or “efficiency.” Conversely, CT increases may reflect edema, inflammation, or myelin/fluid shifts rather than purely neuroplastic remodeling. Without a control group and without fluid-sensitive sequences (e.g., FLAIR) or diffusion imaging (e.g., DTI), we cannot distinguish edema resolution from structural remodeling. (4) Activity-dependent versus compensatory plasticity. Smaller structural change in a given region may reflect a ceiling on local plasticity, engagement of compensatory networks elsewhere, or a shift toward more automatic/less effortful processing—each of which would produce different brain–behavior relationships. (5) Measurement and methodological factors. Test–retest variability of CAT12 surface metrics, spatial smoothing with different kernels across measures (15 mm for CT vs. 20 mm for FD, GI, and SD), partial volume effects at the pial surface, and the small sample size (*N* = 9) can all contribute to negative correlations. In addition, and most importantly, the observed morphometric changes themselves cannot be assumed to represent beneficial neuroplasticity; the negative direction of these correlations therefore cannot be interpreted as evidence that “less change is better”, nor as evidence of a single mechanism such as “neural efficiency.” Because the sample size (*N* = 9) provides low statistical power for correlation analyses, and because the analyses were not corrected for multiple comparisons across the 122 exploratory tests, these relationships should be interpreted as hypothesis-generating only. The Pearson and Spearman coefficients were consistent in direction and significance, but the confidence intervals are wide, and these correlations cannot be taken as evidence of a reliable brain–behavior relationship. Future studies with larger, independently recruited samples, pre-registered correlation analyses, and multimodal imaging (e.g., FLAIR, DTI, and quantitative MRI) are needed to distinguish among these alternative mechanisms. The observed behavioral improvements may be linked to compensatory effects in other brain regions, but this hypothesis requires further study.

The present study had several limitations. First and most importantly, the parent trial was a single-arm, open-label study; all participants received combined TLNS plus physical therapy, and no untreated, physical-therapy-only, or sham-stimulation arm was included. The observed pre–post changes therefore cannot be attributed to the stimulation component, to physical therapy, to their combination, or to other factors associated with repeated assessment (e.g., practice effects, test–retest variability, scanner/preprocessing variability, or natural longitudinal change). Second, the present analysis is restricted to baseline and the end of the 2-week in-lab phase because these were the only time points with both behavioral and usable T1-weighted MRI data for all nine participants; the trajectory of the morphometric and behavioral changes beyond this period, including the 12-week at-home phase, cannot be characterized. Third, this is a secondary analysis of a completed trial (NCT02158494) with *N* = 9; the sample size was determined by the parent trial and was not based on an *a priori* power calculation for surface morphometry, and the analyses were not pre-registered. Fourth, individual-level severity data (GCS, LOC, AOC, PTA) were not retained, so the cohort cannot be separated into formal mild versus moderate categories, and “mmTBI” is used as a descriptive label. Fifth, peripheral vestibular function was not systematically assessed and was not an exclusion criterion, so undetected peripheral vestibular deficits remain a potential confound. Sixth, spatial smoothing followed CAT12 recommendations (15 mm FWHM for CT; 20 mm FWHM for FD, GI, and SD), and the different kernels should be kept in mind when comparing the spatial extent of findings across measures; test–retest reliability of CAT12 surface metrics in TBI populations is also not well established. Seventh, the correlation analyses were exploratory, restricted to regions showing significant group-level morphometric change, underpowered at *N* = 9, and uncorrected for multiple comparisons across 122 tests; they should be interpreted as hypothesis-generating only, and the alternative explanations for the negative brain–behavior relationships are discussed in detail in the Discussion. Finally, the parent trial was open-label and unblinded, and the present analysis did not include a healthy control group or data on comorbidities, medication changes, or concurrent therapies. Changes in cortical surface morphometry in either direction cannot be assumed to represent beneficial neuroplasticity. These findings should therefore be regarded as preliminary, and randomized, sham-controlled trials—ideally with a physical-therapy-only arm, larger independently recruited samples, pre-registered analyses, multimodal imaging, objective vestibular testing, and complete per-participant severity documentation—are needed to determine whether PoNS®/TLNS produces cortical structural changes beyond physical therapy, natural longitudinal variation, and measurement variability.

## Conclusion

In this exploratory, single-group pilot study, cortical surface morphometric changes were observed from baseline to the end of the 2-week in-lab phase following the combined TLNS plus physical therapy intervention and paralleled improvements in balance and gait in mmTBI patients; data from the subsequent 12-week at-home phase and follow-up assessments were not included in the present analysis. Because no untreated, physical-therapy-only, or sham-stimulation comparison group was included, these findings cannot establish causality, and it is not possible to determine whether the observed changes were attributable to the stimulation component, to physical therapy, to their combined effects, or to other factors associated with repeated assessment. Moreover, changes in cortical surface morphometry in either direction cannot be assumed to represent beneficial neuroplasticity; alternative and non-mutually exclusive explanations include focal edema resolution, partial volume and segmentation effects, test–retest measurement variability, and compensatory rather than restorative plasticity. These results should therefore be regarded as hypothesis-generating, and they support the need for randomized, sham-controlled trials—ideally including a physical-therapy-only arm and multimodal imaging (e.g., FLAIR, DTI, and quantitative MRI)—to determine whether PoNS®/TLNS produces cortical structural changes beyond physical therapy, natural longitudinal variation, and exercise-related effects, and to further evaluate the combined intervention as a potential neuromodulatory approach for movement and balance deficits following TBI.

## Data Availability

The raw data supporting the conclusions of this article will be made available by the authors.
